# Fasting-Induced Changes in Serum Kynurenines Do Not Always Reflect Their Urinary Excretion

**DOI:** 10.3390/nu18040689

**Published:** 2026-02-20

**Authors:** Zuzanna Margas, Andżelika Borkowska, Konrad Kowalski, Ulana Juhas, Joanna Reczkowicz, Jakub Kortas, Anna Pilis, Inga Cytrych, Ewa Ziemann, Jędrzej Antosiewicz

**Affiliations:** 1 Department of Bioenergetics and Physiology of Exercise, Medical University of Gdańsk, 80-211 Gdańsk, Poland; zuzanna.margas@gumed.edu.pl (Z.M.); konrad.kowalski@masdiag.pl (K.K.); ulana.juhas@gumed.edu.pl (U.J.); joanna.reczkowicz@gumed.edu.pl (J.R.); jedrzej.antosiewicz@gumed.edu.pl (J.A.); 2 Masdiag Laboratory, 33 Stefana Żeromskiego St., 01-882 Warsaw, Poland; inga.cytrych@masdiag.pl; 3 Department of Health and Life Sciences, Gdansk University of Physical Education and Sport, 80-336 Gdańsk, Poland; jakub.kortas@awf.gda.pl; 4 Department of Health Sciences and Physiotherapy, Jan Długosz University in Częstochowa, 42-200 Częstochowa, Poland; a.pilis@ujd.edu.pl; 5 Department of Athletics, Strength and Conditioning, Poznan University of Physical Education, 61-871 Poznan, Poland; ziemann@awf.poznan.pl

**Keywords:** kynurenine metabolites, picolinic acid, anthranilic acid, xanthurenic acid, quinolinic acid, exercise, urine

## Abstract

Background: The effects of fasting on serum kynurenines (KYNs) have been reported; however, no data are available on whether fasting also modifies their urinary excretion. Kidney organic anion transporters are involved in KYNs excretion, suggesting that changes in serum levels may result from altered urinary elimination. Considering the important role of KYNs in regulating various physiological processes, it is crucial to understand the factors that determine their blood concentrations. The present study aimed to determine the effect of an 8-day fasting period on the concentrations of KYNs in both serum and urine. Methods: Thirteen participants underwent an 8-day fast. The exercise test was performed at baseline after an overnight fast and after 8 days of fasting. Results: Fasting increased the serum concentrations of 3-hydroxykynurenine (3-HK), anthranilic acid (AA), picolinic acid (PA), kynurenic acid (KYNA), and xanthurenic acid (XANA). Conversely, serum kynurenine (KYN) and quinolinic acid (QA) decreased, while 3-hydroxyanthranilic acid (3-HAA) remained unchanged. In urine, KYN, 3-HK, XANA and QA increased after fasting, whereas AA and PA did not change. Conclusions: In conclusion, these findings indicate that fasting generally increases serum kynurenines (KYNs), which are associated with enhanced urinary excretion, suggesting that fasting may stimulate their synthesis. In the case of anthranilic acid (AA) and picolinic acid (PA), their increase in serum does not influence their urinary excretion. Conversely, a decrease in serum KYN and quinolinic acid (QA) may result from enhanced urinary excretion.

## 1. Introduction

Kynurenines (KYNs), which are metabolites of tryptophan (Trp), play a crucial role in regulating various physiological functions, including cell energy metabolism, immune response, neuronal excitability, and the balance of oxidants and antioxidants [1,2,3]. Dysregulation of Trp metabolism mainly manifests as an increased kynurenine (KYN) serum concentration and increased ratios of KYN/Trp and kynurenic acid (KYNA) to 3-hydroxykynurenine (3-HK) (KYNA/3-HK) [4], and it has been linked to many diseases, including depression, diabetes, neurodegeneration, pulmonary arterial hypertension, and dementia [5,6,7,8]. As mentioned above, kynurenines play a crucial role in various processes; however, excessive increases in certain ones have been shown to induce deleterious changes. For instance, KYN enhances the inflammatory response in the brain via quinolinic acid (QA), which can induce oxidative stress [9]. Indeed, it can be anticipated that, in certain instances, alterations in the serum concentration of a specific kynurenine may constitute an adaptive response to a pathological condition process. Conversely, experiments using a pharmacological inhibitor of indoleamine 2,3-dioxygenase (IDO), an enzyme responsible for KYN formation, led to reduced inflammation in COVID-19 infection [10]. This suggests that some kynurenines may exacerbate pathological processes. Thus, understanding the factors that can influence changes in kynurenine metabolism is crucial for understanding pro-healthy interventions, such as fasting and exercise. Fasting for health is well documented and has been shown to induce several beneficial changes that can reverse disease processes or reduce disease risk. Recently, two studies have shown that fasting leads to significant changes in Trp metabolism and that multiple kynurenines increase in serum [11,12]. At the same time, a limited number of kynurenines have been reported in the serum following this procedure. Thus, the primary objective of this study was to determine whether changes in serum kynurenines correlate with changes in urinary excretion after 8 days of fasting. We hypothesize that an increase in serum kynurenines could result from both enhanced kynurenine formation and altered renal elimination. To further assess whether these changes reflect true metabolic adaptation rather than passive accumulation, we measured kynurenine metabolites in response to a single bout of exercise with progressively increasing intensity, performed both before and after the 8-day fasting period. Prolonged fasting is known to induce multiple adaptive responses across human tissues, including skeletal muscle, notably through the upregulation of PGC1alpha, which controls the expression of kynurenine aminotransferase (KAT) and mitochondrial biogenesis [9,13]. Because physical exercise serves as a significant physiological stimulus for muscle-derived kynurenine metabolism, the post-fasting exercise test was conducted to functionally assess whether fasting-induced adaptations resulted in altered kynurenine flux under metabolic stress.

## 2. Materials and Methods

### 2.1. Characteristics of the Subjects

Eighteen healthy males were recruited for the study (average age 61.50 ± 11 years). The cohort included three newly recruited participants, while the remaining individuals had prior experience with fasting interventions, some of whom had undergone this procedure annually for several years, with fasting durations ranging from 3 to 42 days. Thirteen individuals completed the intervention. Blood samples were collected from all 13 participants, and urine samples were obtained from 12 participants. Before commencing the study, participants underwent medical screening. The inclusion criteria were identical to those in the previously published research [11]. Before starting the main experiment, all subjects underwent medical examinations and were deemed qualified to participate. The exclusion criteria included any chronic diseases, smoking tobacco products, medication intake, use of strong stimulants or psychoactive substances, and failure to complete the fasting protocol. Participants were instructed to maintain their habitual dietary patterns throughout the study and were characterized by daily moderate-intensity physical activity, as monitored via mobile applications, with an average of 10,500 ± 2300 steps per day.

### 2.2. Study Design and Ethical Approval

The study involved a short-term dietary intervention (fasting) conducted in a small group of healthy adult volunteers and was designed as an exploratory physiological investigation rather than a clinical trial to evaluate therapeutic efficacy or health outcomes. The study protocol was designed to coincide with the fasting period of the recruited participants.

All participants were fully informed about the study’s aims, methods, and potential risks and provided written informed consent prior to participation. The study was conducted in accordance with ethical standards and international guidelines for research involving human subjects and followed the principles of the Declaration of Helsinki—Ethical Principles for Medical Research Involving Human Subjects. Ethical approval was obtained from the Committee for Ethics in Scientific Research of Jan Długosz University in Częstochowa, Poland (approval no. KE-0/9/2024).

### 2.3. Study Intervention

At baseline, all subjects remained under medical supervision for 3 days prior to the start of the experiment. No alcohol consumption, medication intake, or elevated physical activity was allowed for 4 days before the project started. A body composition assessment and an exercise test to exhaustion were performed. For the next 8 days, participants fasted and drank only water with an average ion concentration. After 8 days of intervention, the same battery of measurements was repeated. Participants were asked to maintain their daily routine, including moderate-intensity physical activity. Consuming caloric meals in any form was forbidden during the examination period.

### 2.4. Anthropometric Measurements

Anthropometric measurement of body composition was taken at baseline and after 8 days of intervention using the bio-electric impedance method (TBF 300A body composition analyzer; Tanita, Amsterdam, The Netherlands). Body mass was measured in a standing position, with participants wearing only underwear. Fat amount expressed in kg and percentage, as well as free-fat mass, were determined. Additionally, body mass index (BMI) was calculated.

### 2.5. Exercise Test

Before and after the 8-day fasting procedure, participants performed an exercise test with increasing intensity until exhaustion on the Excalibur Sport cycle ergometer (Lode B.V., Groningen, The Netherlands). The initial workload started with 60 W and was increased incrementally by 30 W every 3 min. Conditions that caused the test to break included the participant’s oxygen uptake reaching a maximum peak or declining; the heart rate not increasing, stabilizing at its maximum level, or declining; and the pedalling rhythm not being maintained.

### 2.6. Sample Collection

Before the experiment, in the resting condition, serum and daily urine output were collected. The same procedure was conducted after the intervention. The blood samples were also taken immediately after the exercise test. Serum was processed using a standardized protocol before chromatographic analysis. After rapid thawing at room temperature, 50 µL of serum was transferred into a 1 mL deep-well polypropylene 96-well plate. Protein precipitation was performed by adding 250 µL of a reagent containing internal standards, followed by vortexing and incubation for 30 min at 1000 rpm.

The samples were then centrifuged at 3000 rpm for 10 min, and 50 µL of the supernatant was transferred to a fresh polypropylene plate. The collected supernatant was dried thoroughly and subjected to derivatization using a 3 M hydrogen chloride solution in 1-butanol (Merck KGaA, Darmstadt, Germany). Subsequently, the dried residue was reconstituted in 100 µL of 0.1% aqueous formic acid (VWR, Radnor, PA, USA) and injected into the chromatographic system for analysis.

### 2.7. LC-MS/MS Analysis

The sample preparation method for both serum and urine samples involves sequential precipitation and derivatization with n-butanol in 3N HCl prior to LC-MS/MS analysis. The sample (50 µL serum or 30 µL urine) was initially combined with 30 µL of an internal standard solution (MeOH:H_2_O, 8:2, *v*/*v*) and 30 µL of a MeOH:H_2_O (8:2, *v*/*v*) solution containing 0.4 M HCl. Next, analytes were released during a 15 min incubation period at room temperature (RT) on an orbital shaker; precipitation was induced with 240 µL of acetonitrile. Following a further incubation and centrifugation period of 15 min, 75 µL of the supernatant was transferred to a fresh Eppendorf tube and subjected to evaporation (N_2_ for 12 min at 55 °C). The dried residue was then subjected to derivatization with 50 µL of n-butanol in 3N HCl at 60 °C for 25 min. Following a second drying process, the dry residue was reconstituted in 60 µL of 0.1% formic acid in water.

The quantitative analysis of kynurenine pathway metabolites was conducted using high-performance liquid chromatography with the tandem mass spectrometry method. This analysis was performed using serum and urine samples, with the metabolites being measured by means of the isotope dilution method.

The panel covered a range of analytes, including 3-hydroxyanthranilic acid (3-HAA), 3-hydroxykynurenine (3-HK), kynurenic acid (KYNA), kynurenine (KYN), picolinic acid (PA), anthranilic acid (AA), xanthurenic acid (XANA), and quinolinic acid (QA). These were measured against corresponding isotopic reference standards, namely 2H4-KYN, 2H5-KYN, 13C215N-3HK, 13C315N-QA, 2H4-PA, 2H4-XANA, 2H3-3HAA, and 13C6-AA. A quantitative analysis was performed on an LC20AC UPLC system (Shimadzu, Kyoto, Japan) coupled with a QTRAP^®^ 5500+ (Sciex, Framingham, MA, USA). Chromatographic separation was conducted at a rate of 0.8 mL/min on a Zorbax Eclipse XDB-C18 column (50 × 4.6 mm, 1.7 μm; Agilent, Santa Clara, CA, USA) in a gradient of acetonitrile and water (VWR, Radnor, PA, USA) with 0.1% formic acid as the mobile phase. The presence of pseudomolecular ions for butylated kynurenine pathway metabolites was monitored in MRM mode in positive ionisation mode using an electrospray (ESI) ion source.

The Analyst^®^ 1.7.1 software was utilised for the collection of raw data, while MultiQuant^®^ 3.0.1 (Sciex, Framingham, MA, USA) was employed for its subsequent processing and quantification. An evaluation of the reproducibility of the kynurenine metabolites assay was performed using urine samples. The study comprised patient samples (Patient 1, Patient 2, Patient 3) at three concentration levels, each of which was replicated four times over a period of four days (48 samples in total). The sensitivity of the determination of kynurenine metabolites in serum, both analytical (LOD) and functional (LOQ), was calculated based on the signal-to-noise (S/N) ratio of individual MRM transitions. The linearity in the dynamic range of the method calibration is described by the regression coefficient within the dynamic range.

### 2.8. Statistical Analysis

Statistica 13.1 software was used for statistical analysis. The results are presented as the mean ± standard deviation (SD). The Shapiro–Wilk test was used to evaluate the homogeneity of dispersion from the normal distribution. The Brown–Forsythe test was used to assess the homogeneity of variance. To achieve homogeneous results, a paired *t*-test analysis was conducted to identify significantly different outcomes. For heterogeneous results, the Wilcoxon signed-rank test was used. In addition, the difference between measurements was calculated (Δ) with 95% confidence intervals (CI). Additionally, we examined the response to a single bout of training before and after fasting. For homogeneous results, the analysis of variance (ANOVA) for repeated measures and a post-hoc Tukey’s test for equal sample sizes were performed to identify significant differences in results. For heterogeneous results, ANOVA, Friedman’s test, and the Dunn–Bonferroni post-hoc test were used. The significance level was set at *p* < 0.05.

## 3. Results

### 3.1. Effect of Fasting on Body Composition

The applied procedure resulted in changes in body composition (Table 1). The 8 days of fasting decreased the total body weight (BW) and body mass index (BMI). The results obtained indicate statistical significance. Additionally, fat tissue expressed in kilograms and as a percentage of BM decreased significantly. Free fat mass (FFM) decreased significantly in response to the intervention.

### 3.2. Changes in Resting Samples of Serum and Urine Kynurenine Metabolites in Response to the 8-Day Fasting

**Figure 1:**
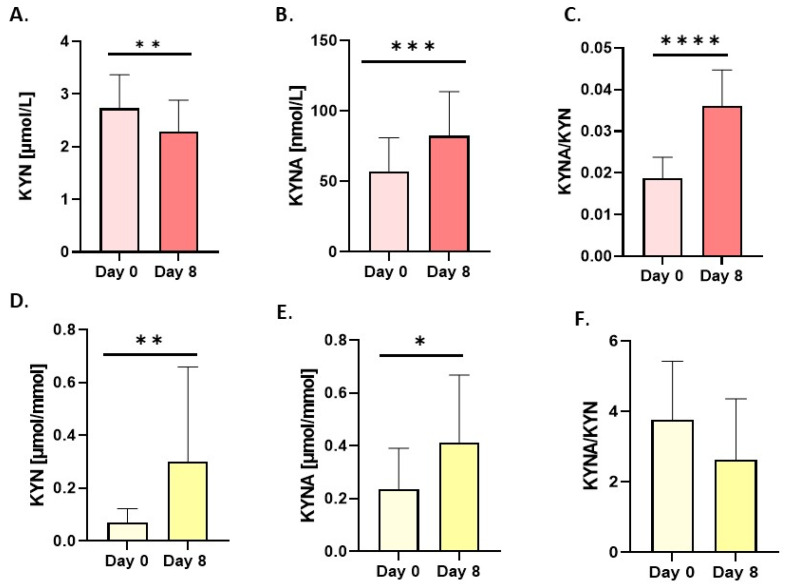
Changes in resting concentrations in serum (red bars) and urine (yellow bars) of (**A**,**D**) kynurenine (KYN), (**B**,**E**) kynurenic acid (KYNA) and (**C**,**F**) ratio of kynurenic acid/kynurenine (KYNA/KYN). Urine values are expressed as µmol/mmol of creatinine. Significant changes are marked with * *p* < 0.05; ** *p* < 0.01; *** *p* < 0.001; **** *p* < 0.0001.

**Figure 2:**
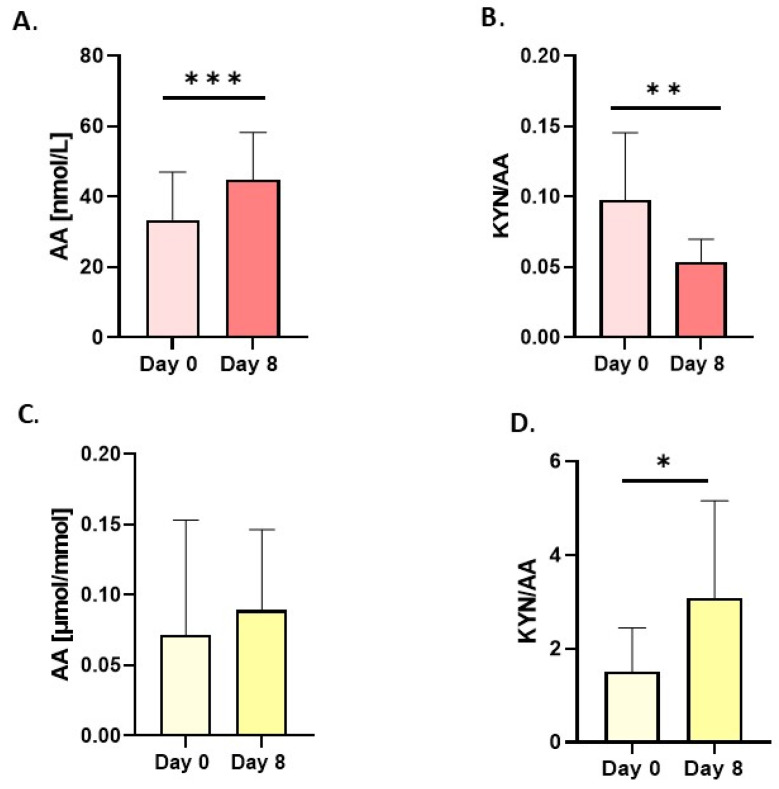
Changes in resting concentrations in serum (red bars) and urine (yellow bars) of (**A**,**C**) anthranilic acid (AA) and (**B**,**D**) ratio of kynurenine/anthranilic acid (KYN/AA). Urine values are expressed as µmol/mmol of creatinine. Significant changes are marked with * *p* < 0.05; ** *p* < 0.01; *** *p* < 0.001.

**Figure 3:**
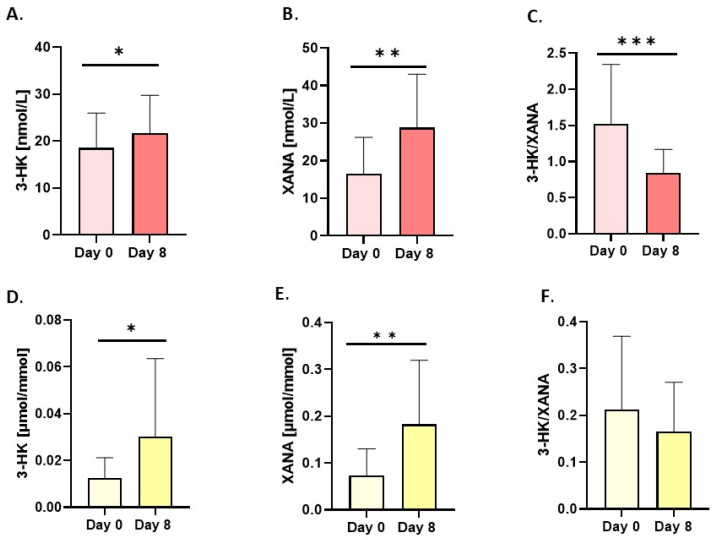
Changes in resting concentrations in serum (red bars) and urine (yellow bars) of (**A**,**D**) 3-hydroxykynurenine (3-HK), (**B**,**E**) xanthurenic acid (XANA) and (**C**,**F**) ratio of 3-hydroxykynurenine/xanthurenic (3-HK/XANA). Urine values are expressed as µmol/mmol of creatinine. Significant changes are marked with * *p* < 0.05; ** *p* < 0.01; *** *p* < 0.001.

**Figure 4:**
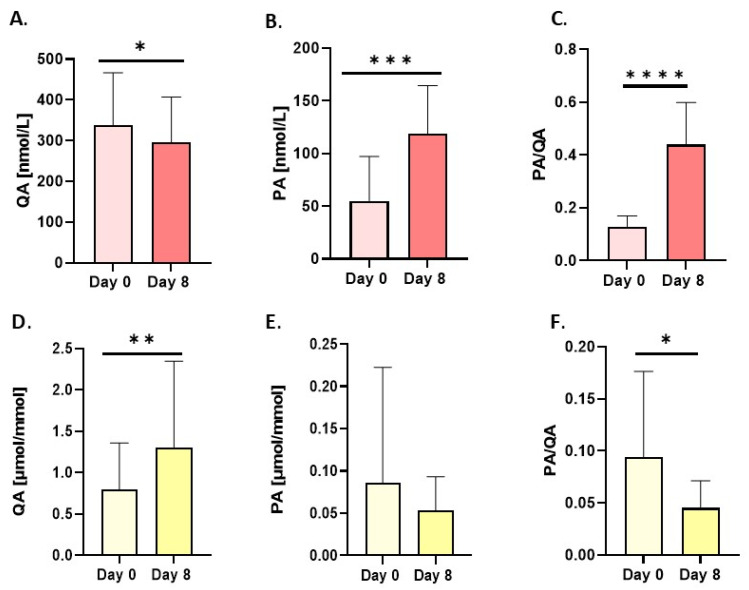
Changes in resting concentrations in serum (red bars) and urine (yellow bars) of (**A**,**D**) quinolinic acid (QA), (**B**,**E**) picolinic acid (PA) and (**C**,**F**) ratio of picolinic acid/quinolinic acid (PA/QA). Urine values are expressed as µmol/mmol of creatinine. Significant changes are marked with * *p* < 0.05; ** *p* < 0.01; *** *p* < 0.001; **** *p* < 0.0001.

**Figure 5:**
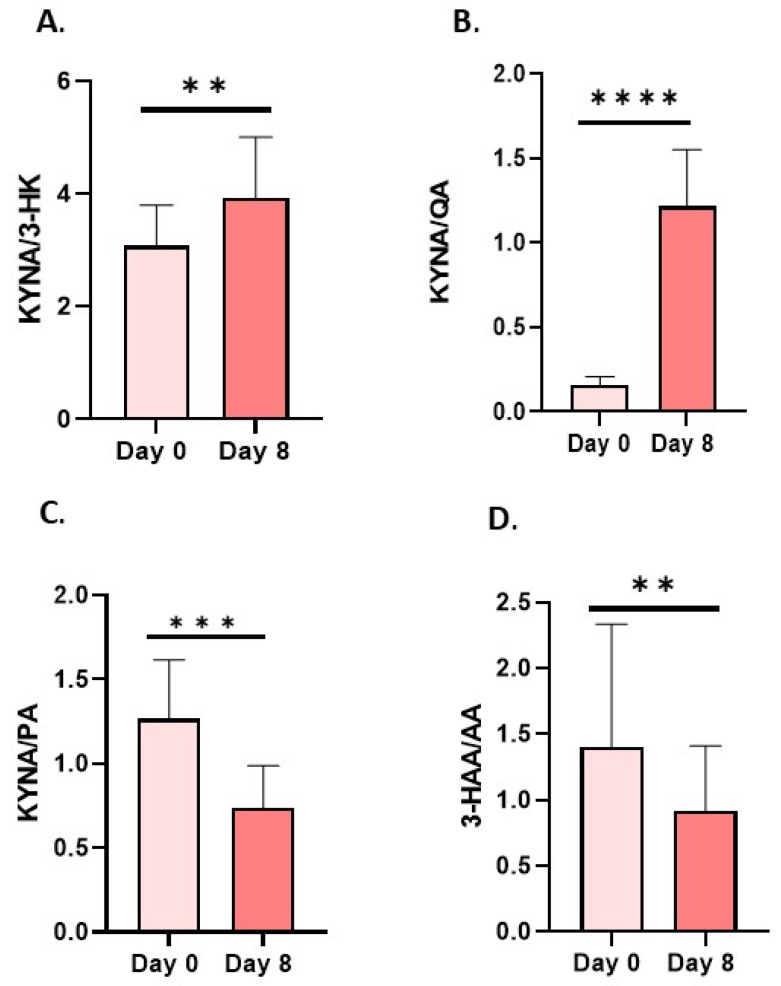
Changes in resting concentrations in serum (red bars) of ratios of (**A**) kynurenic acid/3-hydroxykynurenine (KYNA/3-HK), (**B**) kynurenic acid/quinolinic acid (KYNA/QA), (**C**) kynurenic acid/picolinic acid (KYNA/PA) and (**D**) 3-hydroxyanthranilic acid/anthranilic acid (3-HAA/AA). Significant changes are marked with ** *p* < 0.01; *** *p* < 0.001; **** *p* < 0.0001.

The results are summarized and presented in the following bar graphs (Figure 1, Figure 2, Figure 3, Figure 4 and Figure 5). The 8 days of fasting significantly increased KYNA levels in both blood (74.7%) and urine (71%) samples (Figure 1B,E). At the same time, a notable drop in serum KYN levels was observed (Figure 1A).

In contrast, the concentration of KYN in urine increased significantly (Figure 1D). Changes in these metabolites, expressed as the KYNA/KYN ratio, were significant only in serum (Figure 1C). Anthranilic acid (AA), which has anti-inflammatory properties, increased significantly only in serum after the intervention period (Figure 2A), whereas in urine, the shifts were insignificant (Figure 2C). Divergent changes in KYN and AA levels resulted in a decrease in the serum concentration (Figure 2B) and an increase in the KYN/AA ratio in urine (Figure 2D).

**Figure 6:**
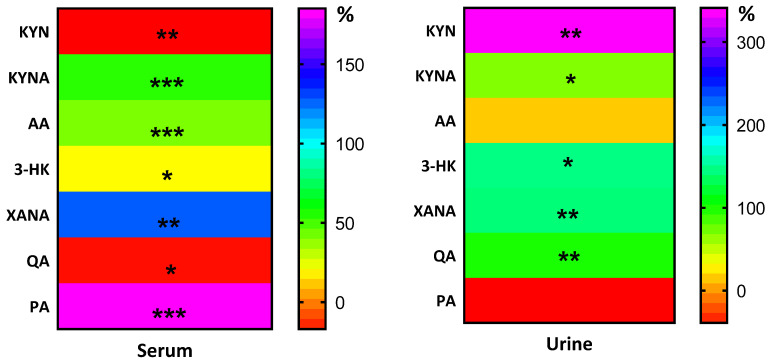
Percentage of change in kynurenine metabolites in blood and urine in response to 8 days of fasting. Significant changes are marked with * *p* < 0.05; ** *p* < 0.01; *** *p* < 0.001.

An indirect reflection of kynurenine aminotransferases (KATs) activity, which generates XANA from 3-HK, is indicated by changes in the ratio of these metabolites, measured in serum. They revealed a higher concentration than baseline (for XANA, an increase of 145.3%) in serum and urine (156%; Figure 3E and Figure 6), whereas 3-HK was elevated by 33.6% in serum (Figure 3A and Figure 6) and 484% in urine (Figure 3D and Figure 6). As a result of changes in the concentrations of XANA and its precursor, 3-HK, the 3-HK/XANA ratio decreased significantly in serum (Figure 3C), whereas in urine this same trend was not statistically significant (Figure 3F). There were no statistically significant changes in either the urine or serum samples of 3-HAA, which showed only minor variations.

Another metabolite of kynurenine that is neurotoxic at high concentrations, quinolinic acid (QA), decreased in serum by 12% after 8 days of the intervention (Figure 4A and Figure 6). Conversely, in urine, QA levels increased by 50% (Figure 4D and Figure 6). At the same time, serum picolinic acid (PA), which has anti-inflammatory properties, increased significantly by 212.9% (Figure 4B and Figure 6), while in urine it decreased by 7% (Figure 4E and Figure 6). Consequently, the PA/QA ratio was significantly higher in serum, while it decreased in urine (Figure 4C,F). Interestingly, an increase in serum KYNA levels led to a significant increase in the KYNA-to-3-HK and KYNA-to-QA ratios, both of which are neuroprotective (Figure 5A,B). Finally, the ratio between KYNA and PA decreased post-intervention (Figure 5C). The ratio between 3-hydroxyanthranilic acid (3-HAA) and AA dropped significantly after 8 days (Figure 5D).

### 3.3. Changes in Kynurenine Metabolites in Serum Induced by a Single Bout of Exercise Performed Before and After 8 Days of Fasting

An 8-day fasting period not only altered resting kynurenine metabolites but also affected the response to exercise. The single bout of exercise increased the concentration of most neuroprotective KYN metabolites, such as KYNA, XANA, AA and PA, when performed post-intervention (Table 2). Consequently, the ratios between those metabolites changed.

## 4. Discussion

We previously demonstrated that an eight-day fasting period significantly modifies the resting concentrations of serum Trp metabolites [11]. In the present study, we confirmed that kynurenines, including KYNA, XANA, and PA, as well as previously untested AA, increased after eight days of starvation. The primary objective of this study was to gain insight into the mechanism by which fasting alters serum levels of kynurenine pathway metabolites. We hypothesize that elevated serum KYNs levels may result from reduced urinary excretion. While kynurenines are known to be excreted in urine and urinary kynurenine levels have been proposed as potential disease biomarkers [14,15], to our knowledge, no study has simultaneously measured serum and urinary KYNs following a fasting period.

In this study, we found that while serum KYN levels decreased after fasting, their urinary excretion increased significantly. Conversely, serum KYNA, a direct metabolite of KYN, increased after fasting, along with its urinary concentration. These findings suggest that the decrease in serum KYN may result from both enhanced conversion to KYNA and other downstream metabolites, as well as increased renal excretion. Because the serum kynurenine-to-tryptophan ratio (KYN/Trp) is commonly used as a marker of IDO activity [16], changes in circulating KYN levels should be interpreted in the context of this ratio rather than KYN concentration alone. Available data indicate that inflammation is another factor that can augment KYN formation, as it increases IDO [17]. In our previous study, we demonstrated that eight days of fasting had no effect on the KYN/Trp ratio, which therefore excludes the involvement of inflammation [11]. In patients with COVID-19, increased inflammation and urinary excretion of KYN and 3-HK have been observed; however, their blood concentrations were not analysed [14]. Importantly, the authors observed no association between KYN and kidney function in patients. Pharmacological inhibition of IDO suppresses SARS-CoV-2-induced proinflammatory cytokine release, supporting a direct role for kynurenines in the exaggerated host immune response. The activity of individual Trp metabolites varies; XANA, KYNA, AA, and, in some conditions, PA have been shown to reduce inflammation, while KYN, 3-HK, and QA can induce inflammation [18,19]. The results of the current study indicate that KYNs with anti-inflammatory properties increase after fasting, whereas proinflammatory KYNs decrease.

Another direct product of KYN metabolism is formed by a reaction catalysed by kynurenine 3-monooxygenase is 3-HK, which can then be converted to XANA by kynurenine aminotransferase (KAT) or to 3-HAA by kynureninase. XANA is possibly not further metabolised; thus, increased serum levels and urine excretion indicate that formation of this metabolite is significantly augmented during the fasting period. Moreover, XANA has been shown to exert neuroprotective and dopamine-stimulating activity, while KYN and 3-HK have some neurotoxic effects [1].

Another pathway that can lead to the formation of 3-HAA is the nonspecific hydroxylation of AA. Here, an increase in the serum concentration of AA with no change in its urinary excretion was observed, while no changes were detected in 3-HAA. A decrease in the 3-HAA/AA ratio has been suggested as a marker of osteoporosis, with values returning to normal after pharmacological treatment [20]. Here, the ratio decreased after fasting, which was due to an increase in AA, and this should be considered a positive change in Trp metabolism. These data are unique, as we are not aware of any study where the effect of fasting on AA has been studied. An elevated blood level of AA has been observed in many clinical conditions [20,21,22]. However, it has been proposed that increasing AA concentration and decreasing the 3-HAA/AA ratio could be a protective response to limit intracellular deleterious changes [23]. In fact, AA has been shown to possess neuroprotective and anti-inflammatory properties [24]. Considering fasting as a pro-health behaviour, the observed increase in AA in serum, together with the absence of change in 3-HAA, should be regarded as advantageous and supportive of previous findings and suggestions. The metabolic fate of 3-HAA is its conversion to PA or QA. Fasting appears to stimulate the formation of PA, as indicated by its increased levels in serum. Conversely, a decrease in serum QA seems to result from its higher urine excretion. Interestingly, urinary excretion of PA did not change despite its high increase in serum. However, it is also possible that the conversion of 3-HAA to PA is augmented, which could explain the lack of changes in serum or urinary 3-HAA. Another possible explanation for fasting-induced changes in urine and serum KYN may involve kidney kynurenine transporters, primarily the organic anion transporters (OATs), such as hOAT1 (SLC22A6) and hOAT3 (SLC22A8), which transport organic anions like kynurenic acid across kidney tubular cells [25]. These transporters play a crucial role in regulating the levels of kynurenine pathway metabolites, including KYNA, KYN, and QA, and in their elimination and reabsorption in the kidney. Interestingly, experiments performed on Oat1 KO mice and in humans treated with probenecid, an OAT inhibitor, showed elevated serum levels of KYN, PA, XANA, KYNA, and several other metabolites, possibly due to reduced elimination [25]. In our study, we observed that after fasting, urinary excretion of KYN, QA, 3-HK, XANA, and KYNA increased, suggesting activation of OAT transporters. However, the elimination of PA, AA and 3-HAA remained unchanged even if serum PA and AA increased significantly. Another factor that can influence KYNs urinary excretion is changes in pH. Here, we observed that urine pH significantly decreased after fasting, which can cause increased excretion of KYNs [26].

Considering that AA can cross the blood–brain barrier [27], an increase in its concentration in the blood after fasting may translate into a higher concentration in the brain. Moreover, AA is an effective inhibitor of 3-HAA dioxygenase, thereby reducing the conversion of 3-HAA to QA [28]. The neurotoxicity of QA is well documented, as it can increase free radical formation and promote excitotoxicity [29]. Thus, it remains probable that, in healthy humans, the conversion of 3-HAA to QA is limited by elevated levels of endogenous AA [23], which can translate to better brain function, as observed after fasting [30]. Moreover, exercise also increased AA formation, while 3-HAA remained unchanged. This is an unexpected observation, as both compounds are products of reactions catalyzed by kynureninase, with the former from KYN and the latter from 3-HK. Interestingly, KYN decreases after exercise, which may reflect its conversion to AA. Conversely, both KYNA and XANA are products of the KAT reaction; thus, their higher levels after the exercise indicate that fasting leads to activation of this enzyme activity, as has been suggested [9,11]. Although exercise-related data may appear peripheral to the primary research question, the existing literature indicates that fasting induces multiple adaptive responses across various human tissues. Notably, fasting has been shown to upregulate PGC1 alpha, which regulates the expression of kynurenine aminotransferase (KAT) activity and increases mitochondrial biogenesis. To verify whether such adaptations were present, we implemented an exercise test with precisely controlled workload intensity. This approach revealed a significantly higher concentration of kynurenic acid, indicating increased KAT activity. Moreover, to our knowledge, this study is the first to demonstrate the combined effects of a single bout of exercise and fasting on serum AA levels.

The present study is not without limitations. First, the relatively small sample size and the inclusion of only male participants limit the generalisability of the findings. Although the inclusion of women would have been of clear scientific interest, a complete fasting intervention constitutes a highly demanding and burdensome procedure. Despite considerable recruitment efforts, we were unable to enrol a sufficient number of women who were willing to undergo this protocol. Consequently, the study was conducted in a volunteer sample consisting exclusively of men, and the lack of sex-based diversity is acknowledged as a limitation.

Second, dietary intake was not strictly monitored prior to the intervention. Participants were instructed to maintain their habitual dietary patterns throughout the study period. Importantly, in our previous investigation [31] which included the majority of participants enrolled in the present study, serum tryptophan concentrations assessed at baseline after an identical standardised fasting period showed negligible inter-individual variability. This finding suggests comparable habitual dietary tryptophan intake across participants and partially mitigates the absence of detailed dietary monitoring.

Third, blood and urine samples were collected at predefined time points rather than daily throughout the fasting period. This design facilitated the evaluation of overall fasting-induced changes; however, it restricts the capacity to observe the temporal dynamics of kynurenine pathway metabolites and renal excretion patterns during the intervention.

These constraints should be considered in the design and analysis of future research.

## 5. Conclusions

In conclusion, this is the first study demonstrating that fasting-induced changes in kynurenines (KYNs) occur in both serum and urine. Interestingly, for KYN, AA, and PA, the changes in serum concentrations induced by fasting were not reflected in urinary excretion. Conversely, a decrease in serum QA appears to result from increased urine excretion. This is an important observation, considering that urinary KYNs have been proposed as markers of disease progression [14,32]. Importantly, these data also indicate that all measured KYNs present in serum are detectable in urine. The effect of exercise on KYNs indicates that fasting induces some adaptive changes manifested by higher formation of anti-inflammatory and neuroprotective kynurenines.

## Figures and Tables

**Table 1:** Anthropometric characteristics of the study group.

Variables	Day 0	Day 8	∆	CI Lower	CI Upper	*p*
**BW [kg]**	88.80 ± 9.48	82.29 ± 9.94	−6.51	−7.20	−5.98	<0.01
**Fat [%]**	23.49 ± 3.46	22.89 ± 3.70	−0.61	−1.79	0.40	0.23
**Fat [kg]**	21.31 ± 5.05	19.35 ± 5.27	−1.96	−3.0	−1.09	<0.01
**FFM [%]**	76.53 ± 3.48	77.12 ± 3.69	0.58	−0.43	1.76	0.24
**FFM [kg]**	67.50 ± 4.84	62.98 ± 4.85	−4.52	−5.79	−3.24	<0.01
**TBW [kg]**	49.41 ± 3.53	46.11 ± 3.55	−3.31	−4.24	−2.36	<0.01
**BMI [kg/m^2^]**	27.93 ± 2.68	25.86 ± 2.81	−2.06	−2.39	−1.90	<0.01

BW—body weight; FFM—fat-free mass; TBW—total body water; BMI—body mass index. Significant changes are highlighted in bold: *p* < 0.05.

**Table 2:** Kyn metabolites in serum in response to a single bout of exercise before and after fasting.

Variables	Day 0	Day 8	∆	CI Lower	CI Upper	*p*
KYNURENINE METABOLITES						
KYN [µmol/L]	2.80 ± 0.49	2.30 ± 0.33	1.45 (−16.5%)	−0.80	−0.21	0.0023
KYNA [nmol/L]	57.98 ± 16.55	97.15 ± 28.51	39.17 (79.47%)	18.87	59.49	0.0006
AA [nmol/L]	32.58 ± 8.22	46.31 ± 9.40	13.73 (28.29%)	7.30	20.17	0.0005
XANA [nmol/L]	16.11 ± 6.60	33.80 ± 10.39	17.69 (153.00%)	9.23	26.15	0.0009
3-HK [nmol/L]	17.58 ± 4.39	21.90 ± 6.60	−12.59 (33.60%)	0.08	8.58	0.0466
3-HAA [nmol/L]	39.59 ± 15.78	36.50 ± 11.97	−3.08 (3.61%)	−13.68	7.50	0.6257
QA [nmol/L]	344.74 ± 113.02	308.03 ± 88.27	−36.71 (−6.28%)	−74.11	0.68	0.0537
PA [nmol/L]	44.47 ± 9.38	118.84 ± 32.57	74.36 (182.38%)	48.13	100.59	<0.0001
RATIO BETWEEN KYNURENINES						
KYNA/KYN	0.02 ± 0.01	0.04 ± 0.01	0.02 (115.00%)	0.01	0.03	<0.001
KYNA/3-HK	3.46 ± 0.94	4.90 ± 1.70	1.44 (42.10%)	0.26	2.62	0.022
3-HK/3-HAA	0.50 ± 0.14	0.64 ± 0.16	0.14 (39.80%)	0.04	0.25	0.011
3-HK/XANA	1.34 ± 0.62	0.71 ± 0.22	−0.62 (−39.00%)	−0.24	−1.01	0.004
3-HAA/AA	1.33 ± 0.62	−9.81 ± 13.28	−10.93 (−1710.55%)	−20.18	−1.68	0.021
KYNA/QA	0.18 ± 0.06	0.33 ± 0.09	0.15 (92.58%)	0.10	0.20	<0.001
KYNA/PA	1.31 ± 0.26	0.86 ± 0.23	−0.45 (−33.11%)	−0.27	−0.64	<0.001
PA/QA	0.13 ± 0.03	0.44 ± 0.13	0.31 (261.60%)	0.22	0.40	<0.001

## Data Availability

The data presented in this study are available on request from the corresponding author.

## Abbreviations

The following abbreviations are used in this manuscript:

KYN	Kynurenine
KYNs	Kynurenines
3-HK	3-hydroxykynurenine
PA	Picolinic acid
KYNA	Kynurenic acid
XANA	Xanthurenic acid
QA	Quinolinic acid
3-HAA	3-hydroxyanthranilic acid
AA	Anthranilic acid
Trp	Tryptophan
IDO	2,3-dioxygenase
KAT	Kynurenine aminotransferase
SD	Standard deviation
CI	Confidence intervals
ANOVA	Analysis of variance
BW	Body weight
BMI	Body mass index
FFM	Free fat mass
BM	Body mass
